# Cost-utility analysis and net monetary benefit of Platelet Rich Plasma (PRP), intra-articular injections in compared to Plasma Rich in Growth Factors (PRGF), Hyaluronic Acid (HA) and ozone in knee osteoarthritis in Iran

**DOI:** 10.1186/s12891-022-06114-x

**Published:** 2023-01-11

**Authors:** Seyed Ahmad Raeissadat, Mohammad Rahimi, Seyed Mansoor Rayegani, Najmeh Moradi

**Affiliations:** 1 Clinical Research Development Center, Shahid Modarres Hospital, Physical Medicine and Rehabilitation Research Center and Department, Shahid Beheshti University of Medical Sciences, Tehran, Iran; 2 Physical Medicine and Rehabilitation Research Center and Department, School of Medicine, Shahid Beheshti University of Medical Sciences, Tehran, Iran; 3 Physical Medicine and Rehabilitation Research Center, Shahid Beheshti University of Medical Sciences, Tehran, Iran; 4 Health Management and Economics Research Center, Health Management Research Institute, Iran University of Medical Sciences, Tehran, Iran

**Keywords:** Knee Osteoarthritis, Cost-utility analysis, QALY, Intra-Articular Injections, Hyaluronic Acid, Platelet-Rich Plasma, Ozone Therapy, Platelet-Rich Growth Factor

## Abstract

**Purpose:**

To evaluate the cost-effectiveness of Platelet Rich Plasma (PRP), Plasma Rich in Growth Factors (PRGF), Hyaluronic Acid (HA) and ozone as effective treatment approaches in knee osteoarthritis management from Iran Health care perspective.

**Methods:**

A decision tree model was conducted to assess the cost-effectiveness of four common intra-articular treatment approaches in patients with mild and moderate knee osteoarthritis. The data on clinical effectiveness was obtained from a randomized controlled trial (RCT) conducted in Iran and used to estimate utility values. The direct medical costs were estimated according to tariffs for public medical centers and hospitals, approved by the Iran Ministry of Health and Medical Education in 2021. The incremental cost-effectiveness ratio (ICER) and the net monetary benefit (NMB) were used to evaluate the cost-utility analysis. Deterministic and probabilistic sensitivity analyses are performed to investigate the robustness of the results and account for the different sources of uncertainty.

**Results:**

In this study, HA intra-articular injection-related costs ($581.67/patient) were defined as the highest cost, followed by PRGF ($328.10/patient), PRP (318.58/patient), and Ozone (103.20/patient). According to the utility value, PRP and PRGF (0.68) have the same and the most utility among Intra-articular injections in knee osteoarthritis management. However, the PRP injection method was identified as the most cost-effective intervention due to its high NMB and ICER estimates. Based on the Monte Carlo Simulation, PR intervention, compared to other ones, was introduced as the dominant strategy regarding knee OA management, with a WTP of $10,000 for 100% of cases.

**Conclusion:**

The study result demonstrated that intra-articular injection of PRP, compare to other injections, is a cost-effective treatment option for patients with mild and moderate knee osteoarthritis. In addition, intra-articular injection of PRP was identified as the best injection, with the highest level of net monetary benefit, for knee OA management.

## Introduction

Osteoarthritis (OA), which is considered one of the leading causes of disability, is a chronic, progressive, and the most common form of joint disorder. Pain, crepitation, stiffness, and decreased range of motion in involved joints, especially knee and hip joints, were reported as OA results [1–3]. Moreover, OA may lead to a decrease in the quality of life and a remarkable financial burden on the economy [4]. The worldwide prevalence of OA is estimated at 35% among people aged 50–59 years, and 55% for people over 70 years [5]. Regarding recent studies, OA is defined as the most common form of arthritis, the sixth cause of disability, and one of the main factors for dysfunction, limitation to job activity, early retirement, and loss of autonomy in older adults worldwide [5, 6].

As we know, there is a vast range of treatment approaches related to OA management, such as non-pharmacological and pharmacological approaches. Non-pharmacological approaches include weight loss, the use of ambulatory aids, exercise, muscle strengthening, and physiotherapy. In contrast, pharmacological treatments include simple analgesics and nonsteroidal anti-inflammatory drugs (NSAIDS). If such treatments would not be effective, therefore, intra-articular knee injections may be considered one of the most efficient non-surgical approaches [7–10]. In addition, surgical treatments, due to their costs and potential risks, are not considered first-line treatment for patients with knee OA [11].

Intra-articular injection of platelet-rich plasma (PRP), intra-articular injection of plasma rich in growth factors (PRGF), intra-articular injection of hyaluronic acid (HA), and intra-articular injection of ozone are defined as Intra-articular knee injections [9, 12–17]. Regarding such injections, preparation methods, length of effect, and costs may differ [4, 9, 15, 18–20]. Regarding the evaluation of OA care costs, a variety of studies have been performed worldwide [1, 3, 4, 6, 21–24]. In a study conducted in the U.S. (1998), it is reported that OA care costs, from the viewpoint of service suppliers, ranged from 5000 to 6000 dollars/patient-years, depending on the patient’s age and disease evolution [1]. Another study was conducted on the lifetime medical costs of knee osteoarthritis management in the U.S. in 2015 [25]. Based on the results, the estimated average lifetime costs for patients who were diagnosed with OA were $140,300. Morover, the results of this study revealed that knee OA non-surgical regimens costs, contrary to surgical treatment like total knee arthroplasty, are low and represent a small portion of all costs for patients with OA.

One of the well-established non-surgical treatment options which have received significant attention from clinicians is Intra-articular injections, which several studies demonstrated their effectiveness. Regarding this, there are number studies that investigate the cost-effectiveness of intra-articular injections. Some of these studies compared two intra-articular injectable options such as PRP and HA [24, 26–28], in most of which PRP was superior to HA. One study only focused on HA and investigates the CEA of a different form of HA in Knee OA treatments. To the best of our knowledge, this is the first study to estimate the cost-effectiveness of four common intra-articular injections in the treatment of knee OA.

## Methods

### Model design

**Fig. 1:**
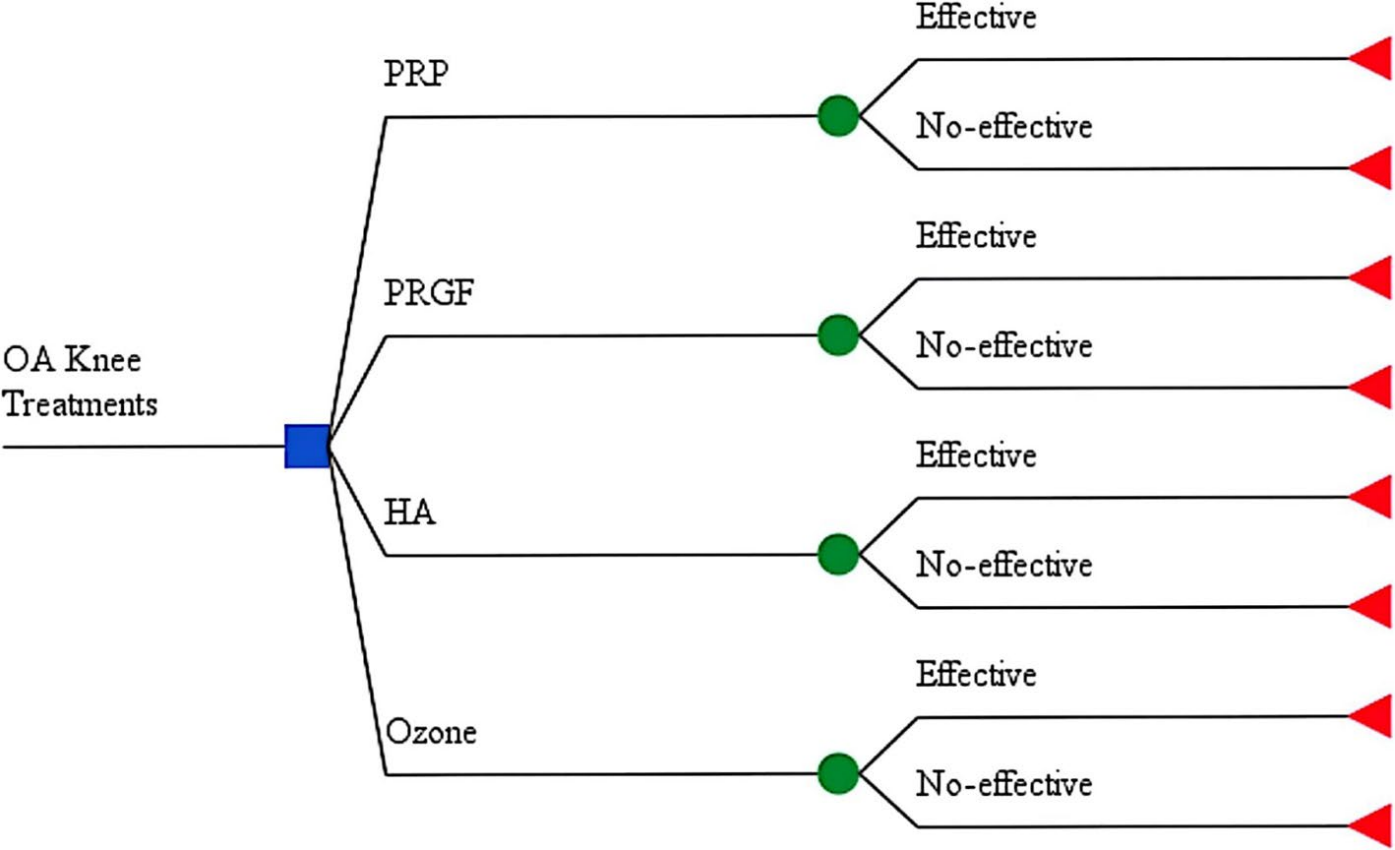
Cost-effectiveness model of Intra-Articular injections

A decision tree model was constructed with four treatment arms to assess the cost-utility of Intra-articular Injections in patients' diagnosed with mild and moderate knee osteoarthritis using Kelgreen Lawrence scale. In one arm, patients would receive PRP as the intervention. The others were comparators including PRGF, HA, and Ozon (Fig. 1).

### Model inputs

#### Treatment utility values

Utility values and probability of success in treatments were extracted from a randomized controlled trial (RCT) conducted by Raeissadat SA et al. [15], which investigated the efficacy of PRP, high molecular weight HA (Hyalgan), PRGF, and Ozone in 200 Iranian patients with mild and moderate knee osteoarthritis in the age range of 40–75 years and with symptoms lasting more than three months, using Western Ontario and McMaster Universities Osteoarthritis Index (WOMAC).

Clinical effectiveness was defined as a positive response to the treatment as more than 70% (i.e., if there was a 30% decrease in WOMAC scores of the intervention in the follow-up period, it is considered effective).

Moreover, to estimate the utility value we use the Wailoo study [29] to convert the WOMAC sub-scales, age, and sex of the patients to the EQ-5D utility value. Finally, the area under the curve method was used to calculate the quality-adjusted life-year (QALY) using the following formula [30].

$$QALY=\sum\limits_{j=1}^nQ_j.t_j$$

#### Treatment cost

In the present study, the health care provider perspective was used to evaluate the costs of intra-articular injection approaches. In this regard, only direct costs, including the cost of products such as kits, the procedures for preparation, and medication-related costs were considered for the analysis, and indirect costs were not included, although their importance (considered the same between different groups in order to make the analysis easier). To estimate the treatments cost we used the medical tariffs for public medical centers and hospitals, approved by the Iran Ministry of Health and Medical Education in 2021. (Conversion rate: 1 dollar equals 42,000 Rials) [31].

### Model analysis

The incremental cost-effectiveness ratio (ICER) and the net monetary benefit (NMB) was used to assess the cost-utility analysis at the WTP threshold value of USD $ 10.000. According to Paulden et al. [32], the NMB is could be considered as an alternative way to assess the economic efficiency of interventions when there are multiple treatment strategies. In this regard, an intervention with an NMB of more than zero is considered a cost-effective treatment and the highest number of the NMB will define the most cost-effective option.

Deterministic sensitivity analysis (DSA) using a tornado diagram was performed to investigate the variation of the ICER/ NMB when parameters are varied independently. The parameters of the model were varied by ± 20 to assess the effect that each variation on the ICER and NMB.

Probabilistic sensitivity analysis (PSA) was performed to investigate the uncertainty of ICER and the possibility of PRP effectiveness. In this regard, a Monte Carlo Simulation approach with 10,000 hypothetical samples was used., Gamma Distribution and Uniform Distribution models were used for cost and utility parameters, respectively.

This analysis was performed using the TreeAge Pro Software (2020).

## Results

### Intra-articular cost of treatments

As shown in Table 1, direct costs related to different intra-articular injections, such as PRP, PRGF, HA, and ozone, were reported. Regarding the results, HA intra-articular injection-related costs ($581.67/patient) were defined as the highest cost, followed by PRGF ($328.10/patient), PRP (318.58/patient), and Ozone (103.20/patient). Moreover, regarding the preparation and injection costs, PRP and PRGF ($68.82/patient) were identified as the highest costs, followed by Ozone ($34.40/patient), and HA ($12.94/patient) (Table 1).

**Table 1:** Direct Costs Related to Intra-Articular Injections

Intervention	Number of Injection	Medication Costs^a^	Preparation and Injection Costs^a^	Single Injection Costs^a^	Total Costs^a^
PRP	2	90.47	68.82	159.29	318.58
PRGF	2	95.23	68.82	164.05	328.10
HA	3	180.95	12.94	193.89	581.67
Ozone	3	-	34.40	34.40	103.20

^a^The costs are based on the conversion of the country's currency into dollar (conversion rate: 1 dollar equals 42,000 Rials)

### Intra-articular effectiveness of treatments

In terms of the utility value, 12 months after the injection, contrary to the baseline value, the utility was increased over time. As shown in Table 2, PRP and PRGF (0.68) have the most and the same utility gain among Intra-articular injections in knee osteoarthritis management.

**Table 2:** The Utility Value Estimated for Intra-Articular Injections

	**PRP**	**PRGF**	**HA**	**Ozone**
Baseline	0.52	0.51	0.51	0.51
After 12 Months	0.68	0.68	0.61	0.58

### Cost-utility analysis

Incremental costs, incremental effects, ICER, and NMB were presented in Table 3. As it is shown, the PRP method, compared to other strategies, was the dominant one (ICER: 7583.16). Whereas, the HA method was the least one related to cost-effectiveness analysis (ICER: -10,871.70). In this study, regarding the NMB analysis, as shown in Table 3, the PRP method was reported as the highest net monetary benefit ($6385.40), followed by PRGF ($6375.88), Ozone ($6316.77), and HA ($5880.31). Therefore, in compared with other intra-articular injections, the PRP injection method was identified as the most cost-effective intervention due to its high NMB and ICER estimates (Table 3).

**Table 3:** Cost-Utility Analysis

Strategy	Cost ($)	Incremental Cost ($)	Effect	Incremental Eff	ICER	NMB ($)
Oz	103.20	0	0.64	0.00	0	6316.77
PRP	318.58	215.38	0.67	0.03	7583.16	6385.40
PRGF	328.10	9.52	0.67	0.00	-	6375.88
HA	581.67	263.09	0.65	-0.02	-10,871.70	5880.31

### Sensitivity analysis

**Fig. 2:**
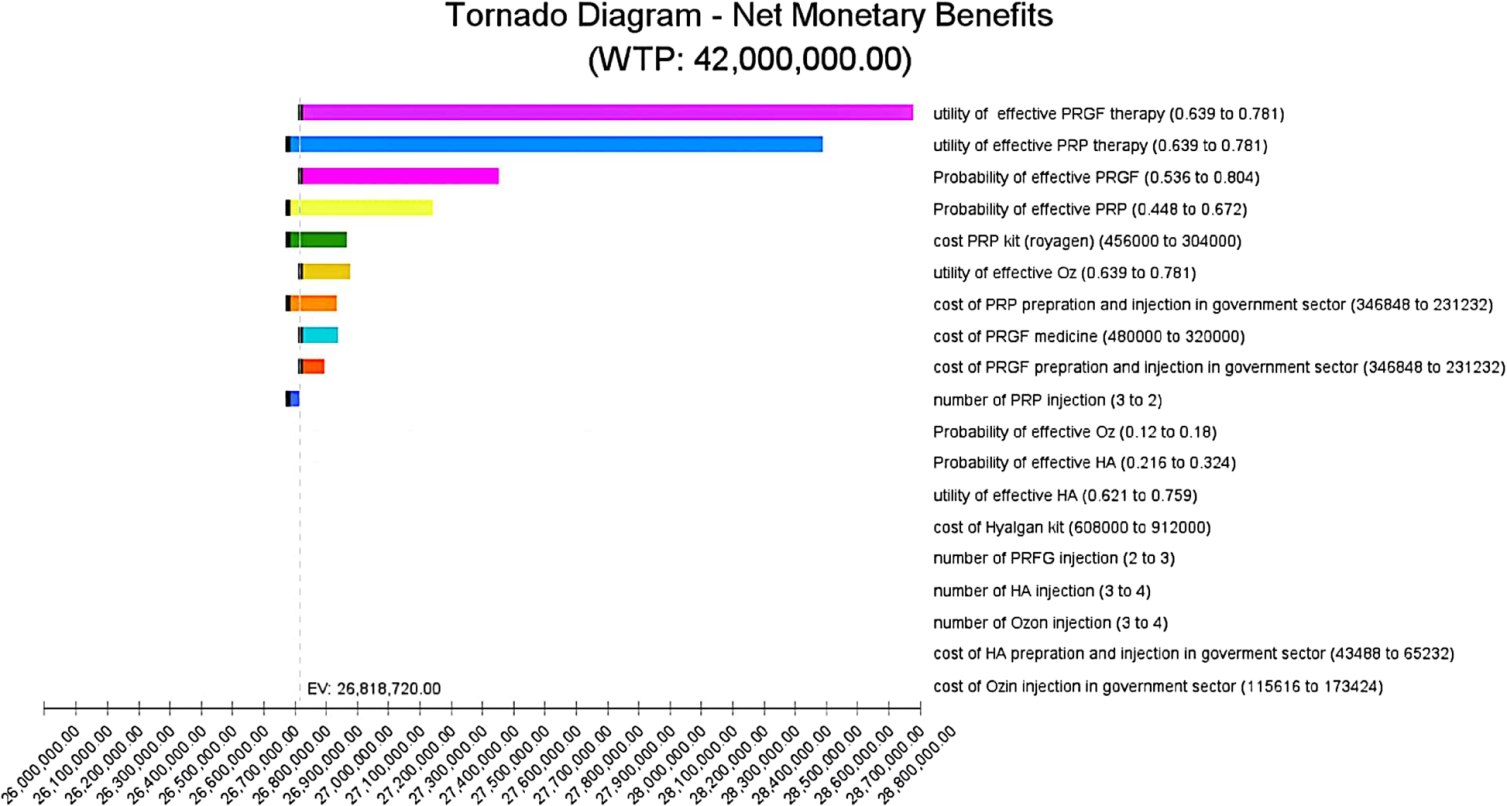
Tornado Diagram

In terms of sensitivity analysis, the Tornado Analysis Diagram and the Monte Carlo Simulation were performed. In this study, the net monetary benefit of PRP, which is defined as the optimal strategy, was considered as the base for comparisons. As shown in Fig. 2, the utility of PRP and PRG alongside the probability of PRP and PRGF effectiveness was reported as the most affected parameters.

**Fig. 3:**
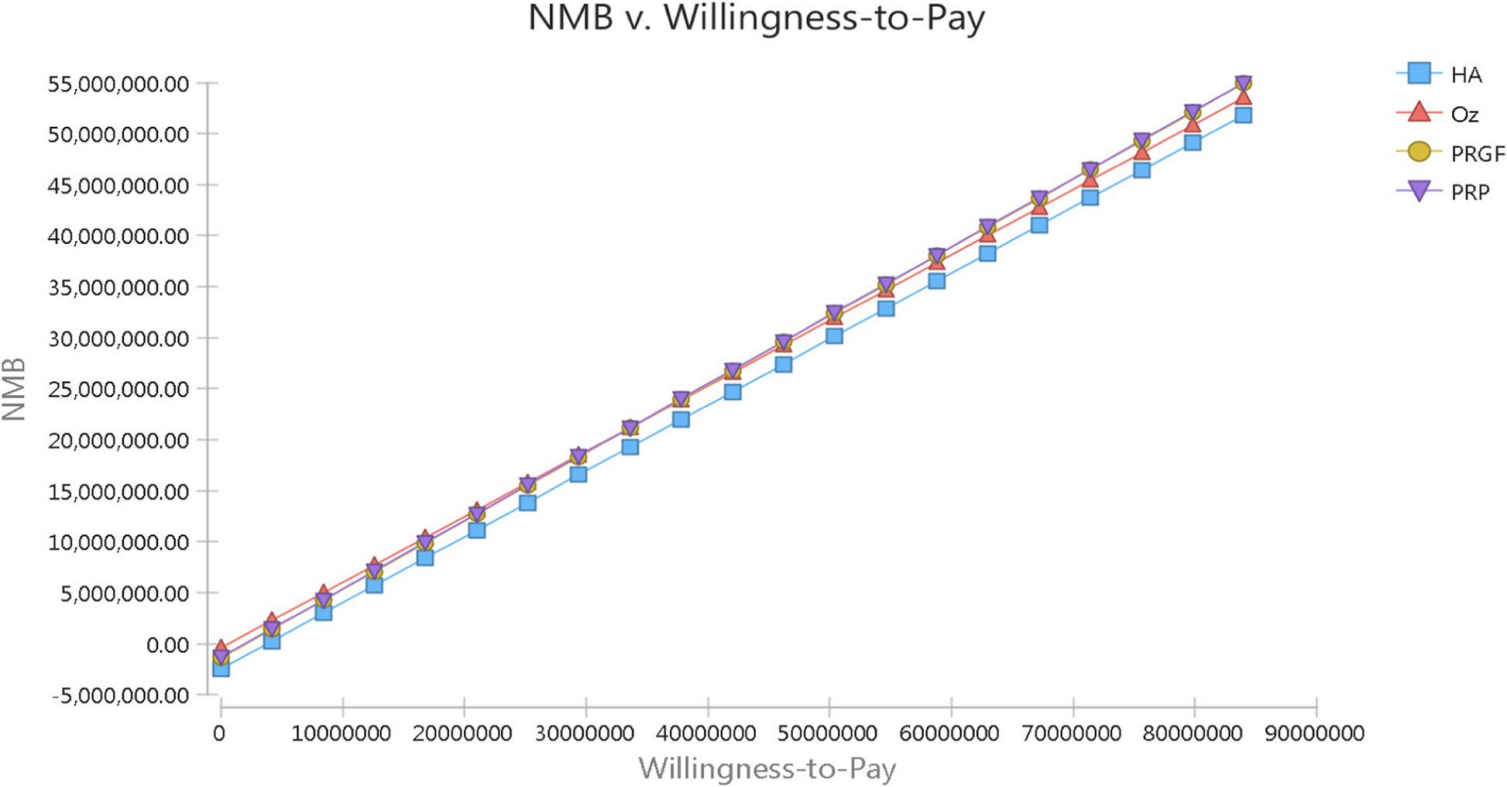
NMB gain in different WTP threshold

We assess the variation in utility of interventions specifically and as it as shown in Fig. 3, by increasing the utility of PRGF by more than 0.71, the cost-effectiveness was changed from PRP to PRGF interventions (Fig. 3). As shown in Table 3, PRP and HA interventions had the highest and the lowest net monetary benefit, respectively. In addition, by increasing the WTP, PRP was introduced as the most profitable intervention, followed by PRGF.

**Fig. 4:**
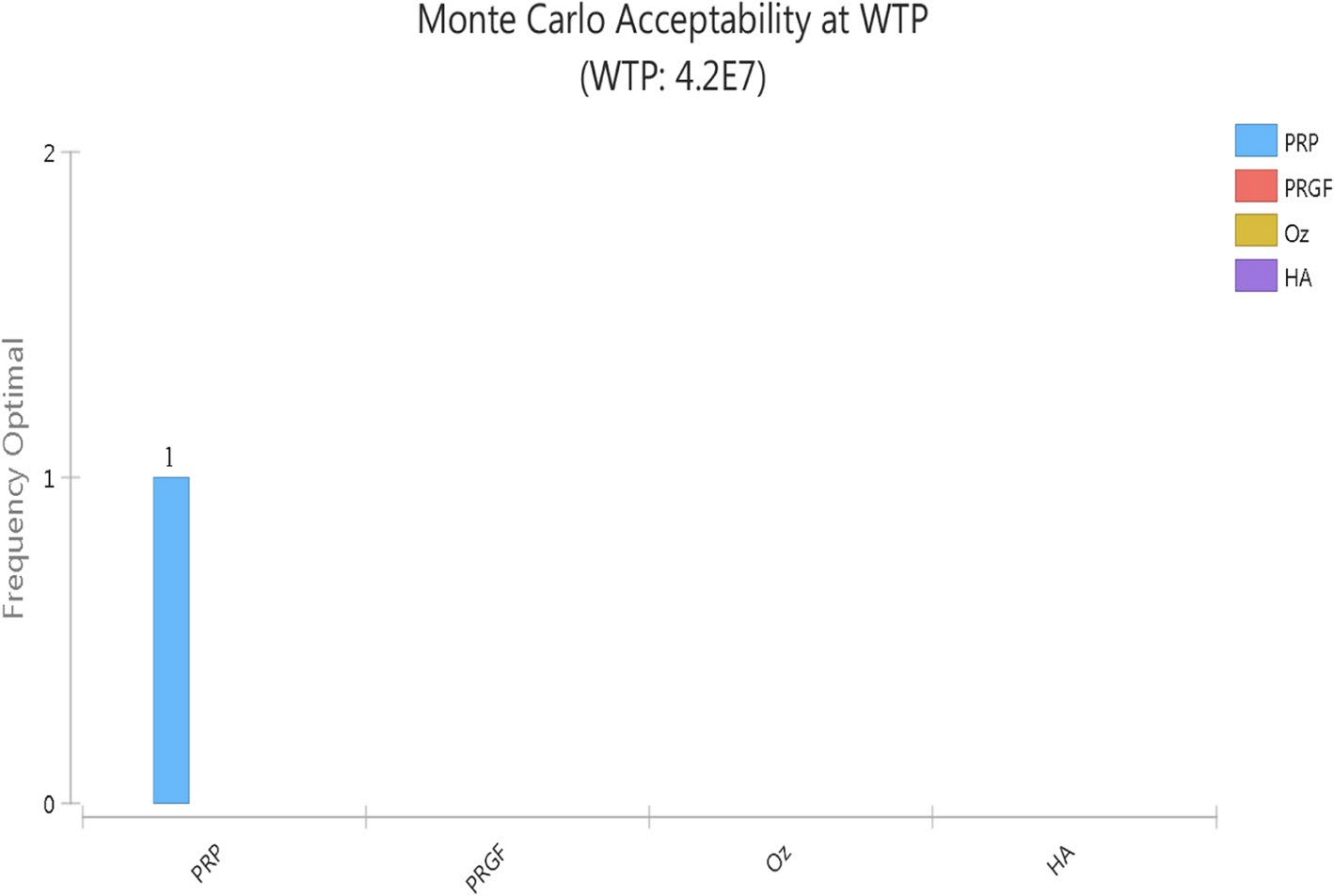
Monte Carlo Simulation chart

Based on the Monte Carlo Simulation, which is shown in Fig. 4, PRP intervention, compared to other ones, was introduced as the dominant strategy in regard to knee OA management, with a WTP of $10,000 for 100% of cases.

## Discussion

Knee OA is a chronic progressive disease in nature and pre-existing nonsurgical treatments have not yet been proven as a curative treatment for this disease. In advanced cases, joint replacement surgery is the gold standard treatment. Therefore, considering the medical complications of such major surgeries, as well as the financial burden of a long recovery period and the need for rehabilitation has led to an increased effort to find alternative treatments. Up to now, several intra-articular injections, such as PRP, PRGF, HA, and Ozone have proven their effectiveness in numerous clinical trial articles and systematic reviews [4, 9, 15, 17–20]. In this regard, the cost-effectiveness comparison of these interventions is one of the missing links in knee OA management.

In recent years, a majority of studies have focused on comparing the effectiveness of injectable and non-injectable interventions regarding knee OA treatment [1, 3, 4, 6, 21–23, 33, 34]. A few studies have also been conducted to compare only two types of intra-articular injections in the treatment of knee OA [13, 24, 26–28]. Due to different socioeconomic, genetic, and environmental parameters in each region, as well as the paucity of a comprehensive agreement on the cost-effectiveness of different intra-articular injections, this study was aimed to evaluate the cost-effectiveness of Intra-Articular Injections in knee Osteoarthritis management. In this study, four types of intra-articular injections, such as PRP, PRGF, HA, and Ozone were studied to evaluate their cost-utility of them regarding knee OA treatment. Based on recent studies, intra-articular injection of PRP would be an effective treatment for patients with knee osteoarthritis [7, 9, 11, 12, 14, 15, 19] as well as a popular choice among the Iranian physicians in the recent years. Therefore, in the present study, we compare the cost-effectiveness of PRP compared to other conventional injections, such as PRGF, HA, and Ozone in knee OA management.

The study results revealed that among intra-articular injections for knee osteoarthritis treatment, PRP, in comparison to PRGF, HA, and Ozone, by acquisition of the most ICER (7583.16), is the most cost-effective injection. In addition, regarding the net monetary benefit, PRP was reported as the best intervention with the highest net monetary benefit (6385.40$). Moreover, based on the sensitivity analysis results, the acceptability of PRP and PRGF, and the probability of efficacy of PRP and PRGF were the most important variables affecting the outcomes of the study (Fig. 2: Tornado Diagram).

The current results are reasonable due to the economic conditions in Iran. High molecular weight HA, used in the original study, was Hyalgan, an importable drug without the cover of insurance, that by considering the lower value of the national currency of Iran, would be an expensive drug (180$), however during last years the preparation kits of PRP as well as PRGF are made in Iran, so though the greater tarrifs for PRP and PRGF injections in medical system in Iran, the total cost of the treatment would be greater in HA group due to the expense of Hyalgan.

Based on our results, we recommend the decision makers in Iran that it would be wise to include the PRP under the insurance coverage, till then if a physician wants to choose between PRP, PRGF, Hyalgan, and Ozone in Iran, the first choice could be PRP on the same situation, considering its cost-utility.

During last year's, scientists in Iran tried to produce high molecular weight HA internally by cheaper means, so comparing the effectiveness as well as cost-utility of these products with the current available HA in Iranian markets, could be considered as the topic of future studies.

## Limitation

One of the main limitations of the present study is the lack of indirect costs calculations like reduced productivity of interventions and transportation costs. In addition, in this study, the costs of common conservative treatment, such as oral or topical analgesics, physical therapy, and medical equipment were not considered in the cost-utility analyses. The costs considered in the present study may differ in different situations. It is due to different provider perspectives, providing centers (public or private), contractual agreements, insurance status, and so on. Based on the mentioned reasons, it appears that we could not interpret the results for the general population exactly. A wide range of variations of the drugs and kits costs, as well as the medical tariffs, over time, which is considered another limitation, the reproducibility of the results would not be precise. To cope with such problems, in the following studies, the researcher may calculate the exact costs of different treatments and procedures.

## Conclusion

The study results demonstrated that intra-articular injection of PRP; compare to other injections would be a cost-effective treatment for patients with mild and moderate knee osteoarthritis. In addition, intra-articular injection of PRP was identified as the best injection, with the highest level of net monetary benefit, for knee OA management.

## Data Availability

All data is available and can be provided by the corresponding author upon rational request.
